# Impact of Mechanical Refining Conditions on the Energy Consumption, Enzymatic Digestibility, and Economics of Sugar Production from Corn Stover

**DOI:** 10.1021/acssuschemeng.3c03796

**Published:** 2023-10-21

**Authors:** Yudong Li, Ryan Davis, Eric C. D. Tan, Jacob Dempsey, Kelsey Lynch, David A. Sievers, Xiaowen Chen

**Affiliations:** † Catalytic Carbon Transformation and Scale-Up Center, National Renewable Energy Laboratory, 15013 Denver West Parkway, Golden, Colorado 80401, United States; ‡ Energy Systems Integration, National Renewable Energy Laboratory, 15013 Denver West Parkway, Golden, Colorado 80401, United States

**Keywords:** lignocellulosic biofuel, pretreatment, sugars, milling, biomass, techno-economic analysis (TEA), life-cycle assessment (LCA)

## Abstract

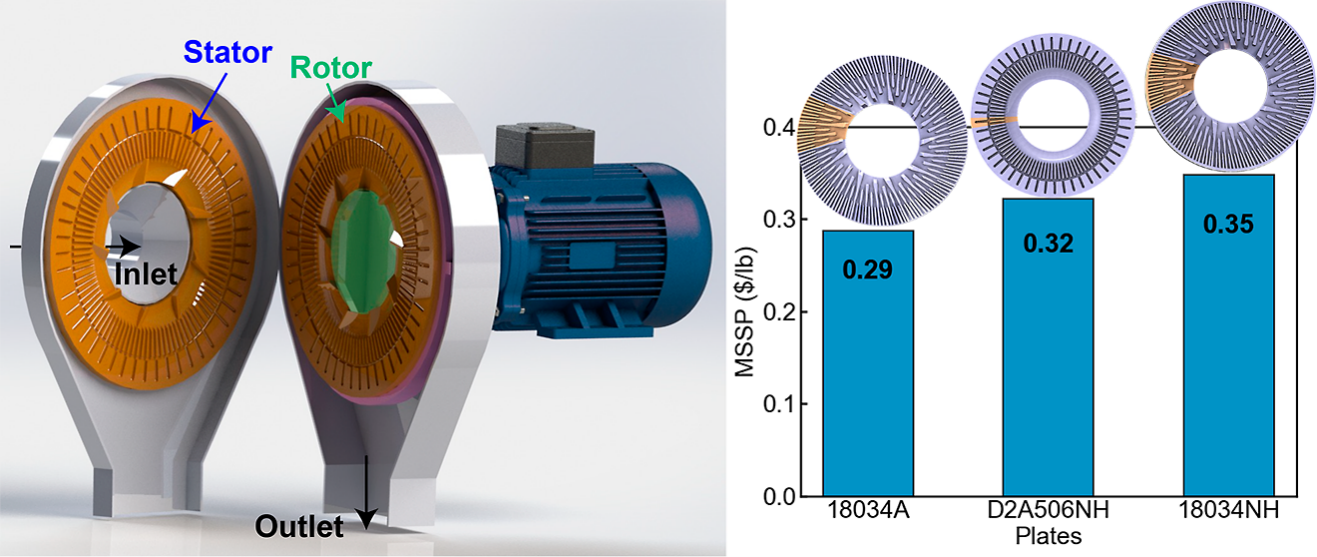

Reducing the energy intensity of the mechanical refining-based pretreatment process for producing lignocellulosic-derived sugars without significantly affecting enzymatic hydrolysis sugar yields is challenging. This work investigated the impact of different refining conditions on energy consumption, enzymatic sugar yields, minimum sugar selling price, and environmental impacts for the conversion of corn stover to sugars. A positive proportionate correlation between specific energy consumption and enzymatic sugar yields was observed when changing the refiner plate gap was changed, which agrees with other reported works. However, the correlation between specific energy consumption and enzymatic sugar yields is not straightforward when the rotational speed and refiner plate design change. We observed that, for a corn stover material with low consistency disc refining, specific energy consumption decreased by >50% by decreasing the rotation speed without affecting enzymatic sugar yields. By changing refiner plate designs, a 45% reduction in specific energy consumption could be achieved without affecting the glucose yield, albeit still with a detrimental impact on the xylose yield. Our high-fidelity disc refining model was able to predict the energy consumption for different refiner plate geometry designs and operating conditions. Techno-economic and life-cycle analyses indicate that the plate design and operating conditions have a direct impact on overall process power consumption and sugar yields, with sugar yields strongly dictating the minimum sugar selling price, the life cycle greenhouse gas emissions, and fossil energy consumption. To minimize the environmental impact and maximize process economics, optimization of the mechanical refining process should target maintaining high sugar yields, while lowering refining energy consumption.

## Abstract 2

Mechanical refining operation conditions significantly affect the sustainability and emission of the conversion of lignocellulosic biomass to sustainable fuels.

## Introduction

To facilitate the deployment of biochemical conversion of lignocellulosic biomass to sustainable fuels and chemicals, the development of environmentally benign and economically viable technologies is critical.^1−3^ The pretreatment process is a critical step in biochemical conversion to increase the renewable carbon utilization. Pretreatment enhances the ability of enzymes to effectively hydrolyze the carbohydrate polymers in lignocellulosic biomass to sugars for further microbial- or chemical-based conversion to other upgradable compounds. The deacetylation and mechanical refining (DMR) process being investigated at the National Renewable Energy Laboratory (NREL) is a nonthermal pretreatment process, which produces biomass with high enzyme accessibility and produces sugar stream with high fermentability/convertibility.^4−7^ The DMR process for converting corn stover includes a dilute NaOH pretreatment step at 92 °C with a solid loading of 8% w/w for 2 h, followed by the dewatering process with a screw press to remove residue black liquor to reach 30–40% w/w consistency for the ease of storage. The pretreated corn stover is then subjected to a disc refining process, which operates at a range of % solids (usually at or below the dewatered corn stover % solids) depending on the specific needs. After disc refining, a pH adjustment is generally required to reach the optimal conditions for enzymatic hydrolysis (EH). Disc refining, as a critical step in the DMR process, increases enzyme accessibility by mechanically disrupting the supramolecular structures of lignocellulosic biomass without introducing microbial inhibitors commonly produced by other pretreatment techniques.^8−10^ For example, dilute acid pretreatment produces sugar degradation products (furfural), high acetic acid concentrations, and lignin-derived phenolics.

The disc refining process is a widely used technology in the pulp and paper industry due to its effectiveness in breaking down woody biomass to fibers.^11^ Many researchers reported studies on disc refining for pulping processes.^12−14^ However, it is also an energy-intensive process. Due to the complexity of refiner geometric features and complex flow properties of fiber suspensions, developing a comprehensive predictive computational model for the disc refining process is challenging. Empirical or semiempirical approaches have been reported^15,16^ in modeling the power consumption as a function of plate gap and other parameters. Empirical models have limitations of broad applicability in different types of refiners with different geometries. A few recent studies^17,18^ started to employ a computational fluid dynamics (CFD)-based approach in modeling the disc refiner with either simplified computational geometric domain or simplified biomass slurry physical properties. Using a high-fidelity geometric computational domain for the disc refiner and accurate biomass slurry rheological properties, our previous work^19^ developed a comprehensive CFD model, which correctly predicted the energy consumption for low-consistency disc refining of corn stover.

While most studies investigate disc refining for pulping applications, a few studies reported using disc refiners to produce woody biomass slurry for enzymatic saccharification. Zhu et al.^20^ studied the energy consumption and enzymatic sugar yields of disc refined and chemically treated lodgepole pine. Their study showed that the EH glucose yield is independent of pretreatment conditions, disc refining solids loading, and refiner gap for sulfite, dilute acid, and hot water-pretreated biomass. They concluded that the disc refining energy intensity is the sole determinator of the enzymatic glucose yield at a majority of their tested experimental conditions. They also observed slight dependence of enzymatic glucose yield on disc refining conditions for low refining energy conditions (low solid consistency and large refiner gap). Jones et al.^21^ performed studies to optimize the disc refining process for the EH of pretreated hardwood biomass. They observed that the enzymatic sugar yield is positively proportional to specific net refining energy regardless of refining solid consistency and refiner gap. Conclusions from these two studies should not be construed to infer that the refining solid consistency and refiner gap do not affect enzymatic sugar yields of disc refined woody biomass. Refining solid consistency and refiner gap affect sugar yields due to their effect on refining energy consumption. A physics-based correlation between the refining conditions and the refining energy consumption is missing in all previously reported works on biomass disc refining prior to EH. The refining energy was used as a lumping variable that affects the enzymatic sugar yields. This is one important reason that many of the prior research works report only refining energy (or refining intensity) without investigating other refining conditions. However, the refining energy is a function of many different aspects of the disc refining process. In a disc refining process, the energy used to break down the lignocellulose supramolecular structure is only a portion of the energy used in the entire process. Energy is consumed to move the slurry (converted to momentum energy) and overcome friction (viscous dissipation and conversion to thermal energy). Thus, the goal of this work is to examine more closely how refining conditions affect enzymatic sugar yields and refining energy consumption.

In this study, we employed both experimental and mathematical approaches to study the disc refining process to further elucidate how disc refining affects the EH yields for herbaceous biomass. In addition to the refiner gap, this work is the first to investigate and report on the effects of refining rotational speed and refiner plate geometry on the sugar yields and energy consumption. We also performed numerical simulations at different refining conditions using our state-of-art disc refiner model reported earlier^19^ and compared energy consumption predictions with experimental measurements. Simulation results were used to elucidate the possible mechanism of how disc refining conditions affect the disruption of the structural integrity. Techno-economic and life-cycle analyses were performed to investigate how changing disc refining conditions would affect process economics and environmental sustainability metrics.

## Materials and Experimental Methods

### Feedstock

Harvested in September 2019 at Hardin County, Iowa, the corn stover with a moisture content of 7% (w/w) was hammer-milled at Idaho National Laboratory (INL) to pass a 2 in. (50 mm) screen. After the corn stover was received at NREL, it was further milled using a knife mill (Mitts and Merrill, model 10 × 12, Saginaw, MI, USA) to pass a 34-in. (19 mm) round hole rejection screen.

### Deacetylation

A 1900 L horizontal paddle reactor was used to perform deacetylation as previously reported by Chen et al.^4^ Corn stover was pretreated at 92 °C for 2 h using a 0.7% (w/w) NaOH solution at a loading of 80 kg NaOH/oven dry metric tonne (ODMT) biomass. The solid consistency was 8% (w/w). The pretreated corn stover slurry was separated using a strainer, and the solids were rinsed once with water to displace the residual liquor. The pH of the slurry was adjusted to 4.6 by adding 95 wt % sulfuric acid. Then, the slurry was dewatered using a continuous screw press (Vincent Corp., model CP10, Tampa, FL, USA) to 30–40% solid consistency.

### Disc Refining

In this study, a 12 in. disc refiner (Sprout Waldren Koppers, model 12″, discontinued by the manufacturer) was used to perform mechanical refining. Biomass slurry after deacetylation was diluted to 3% (wt) total solids and then refined using three different refiner plates at various rotational speeds and plate gaps, shown in Table 1. Refiner plates were installed on both the rotor and stator, where the plate on the rotor rotates against the static plates installed on the stator. Refiner plates 18034A, 18034NH, and D2A506NH with different bar-groove geometric structures are used, as shown in Figure 2. The refiner is controlled by a variable-frequency drive (VFD) that allows for adjustment of the refiner’s rotational speed. The VFD is connected to a supervisory control and data acquisition system (SCADA), which monitors and records the operating conditions and energy consumption. Idle energy consumption was measured by running the disc refiner without biomass slurry at various rotational speeds (Ω_R_ in revolutions per minute, rpm). The idle power correlation as a function of rotational speed is expressed in eq 1, as reported in our previous study,^19^ with α = 5.5 × 10^–5^ and β = 1.4. The total energy consumption (*P*_total_) is the summation of net refining energy consumption (*P*_net,*i*_ with *i* = *experiment* or *simulation*) and idle energy consumption, as shown in eq 2. The total specific energy consumption (*e*_total_) of disc refining at a certain biomass slurry feed rate (*ṁ*_b_) is calculated using eq 3.
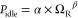
1
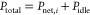
2
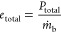
3

**Table 1:** Experiment Conditions Used in Disc Refining as Well as Simulations

case	plate	gap (1/1000 in.)	rotation speed (rpm)
1	18034A	10	900
2	18034A	15	900
3	18034A	20	900
4	18034A	10	600
5	18034A	10	1200
6	D2A506NH	10	900
7	18034NH	10	900

### Enzymatic Hydrolysis

Enzymatic digestions of washed pretreated residues from the pretreatment experiments were performed in 125 mL Erlenmeyer shake flasks at a 1% cellulose solids loading (approximately 2 wt % solids loadings) in a shaking incubator at 50 °C and 130 rpm. Novozymes CTec3 enzyme preparation was added at the level of 12 mg per gram of cellulose with the additional HTec3 enzyme at 3 mg per gram of cellulose. Sodium citrate buffer (1 M, pH 5.3) was added to maintain the pH. Small doses of tetracycline were added as antibiotics. The total volume of the saccharification slurries after adding enzyme and sodium citric buffer was 50 mL. The hydrolysis was terminated at 120 h, and the final sugar concentrations were measured following the NREL standard Laboratory Analysis Procedure^22^ for sugar and byproducts and used to calculate glucose and xylose yields from cellulose and xylan, respectively.^23^ All EH experiments were performed in duplicates. The error bars in sugar yield figures in this article represent the variance of sugar yields of these duplicated experiments.

### Rheology Properties Measurement

**Figure 1:**
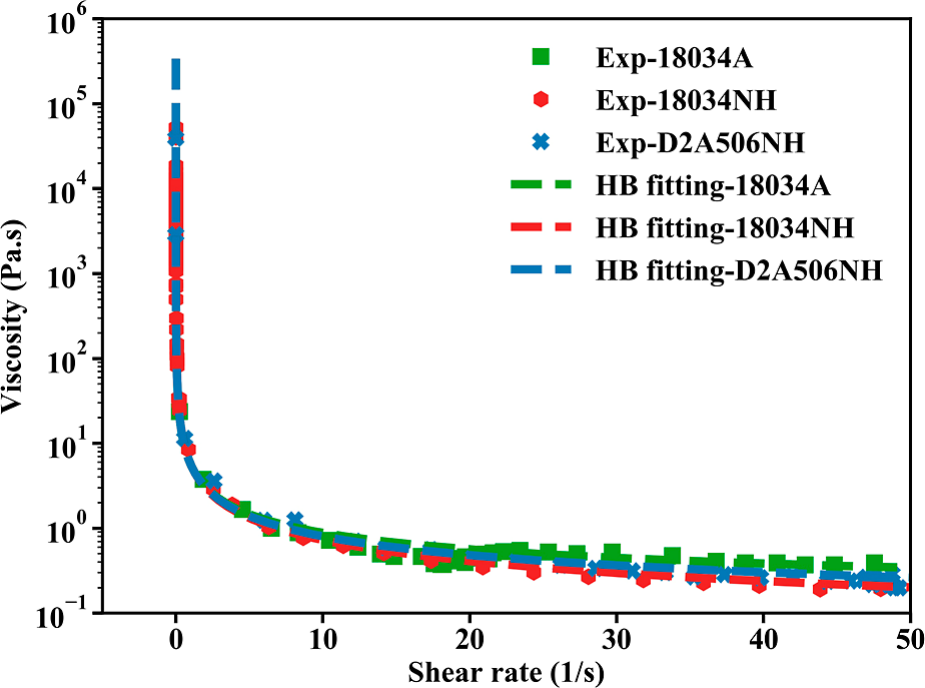
Biomass slurry viscosity correlation with the shear rate obtained from rheometer measurement (blue squares) and the Herschel–Bulkley modeled result (line).

Accurately describing the shear rate of the biomass slurry in response to the shear stress is critical in developing a high-fidelity model for the simulation of the disc refining process. Biomass slurry processed in the disc refiner equipped with different plate geometries (18034A, 18034NH, and D2A506NH) was collected. Rheological properties of these biomass slurry were characterized using a Bohlin Gemini HR Nano rotational rheometer (Malvern Instruments, Westborough, MA, USA). The Herschel–Bulkley model (eq 4) was used to fit the rheology measurement results for biomass slurries. The viscosity (μ) as a function of shear rate for biomass slurries produced using different plate geometries is then calculated by using eq 5, which is shown in Figure 1.
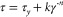
4
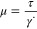
5

### Techno-Economic Analysis

To assess the economics of this process based on the different plate types, plate gaps, and rotational speeds, techno-economic analysis (TEA) models for lignocellulosic sugar production published by NREL were utilized^24^ using data reported here for deacetylation conditions (80 kg sodium hydroxide/dry tonne biomass), enzyme loadings (15 mg/g of cellulose), sugar yields (glucose and xylose, while arabinan-to-arabinose conversion in EH was fixed at 51% per prior unpublished work at similar conditions), as well as specific disc refining energy (kWh/dry metric tonne). The capital cost of the mechanical refining equipment was assumed to remain constant regardless of the specific energy use. The disc refiner capital costs are based on vendor-supplied quotations utilizing a series of parallel disc refiners 54 in. in diameter processing delivered biomass at a 1/4 in. particle size as fed to the deacetylation reactor following upstream preprocessing/milling operations (outside the scope of the biorefinery conversion TEA models); rather, the delivered feedstock cost targets a value of $71.26 per dry ton inclusive of all preprocessing operations.^25^ The TEA model also applies a fixed consistency of 20% of total solids fed to disc refining. While this is higher than the more dilute experimental conditions evaluated at a laboratory scale in this study, we have demonstrated in a prior work the ability to maintain consistent sugar yields spanning such disc refining solids concentrations.^26^ Mechanical refining power demands were originally based on 200 kW h per dry tonne of biomass feedstock processed, reflecting the abovementioned design details (scaled accordingly in this study over varying disc refiner scenarios).

The model utilized to generate inputs for TEA was built in Aspen Plus based on a plant producing concentrated sugars (approximately 50 wt %) from corn stover at a delivered feedstock rate of 2000 dry metric tonnes per day processed through pretreatment. The plant is assumed to operate for 7884 h per year (90% on-stream time) for 30 years. The modeled process operations include deacetylation and mechanical refining pretreatment, with the resulting solids subjected to EH utilizing a mixture of cellulase and hemicellulase enzymes (modeled as being produced on-site using externally purchased glucose). The hydrolysate sugar product is clarified to remove solids using a vacuum filter press, followed by concentration to roughly 650 g/L total sugars using a vacuum mechanical vapor recompression evaporator. Deacetylation black liquors are routed to wastewater treatment (included on-site), while lignin and other residual solids and process off-gases are combusted in a boiler for steam and power cogeneration. The resulting generated power is used to offset facility power demands, in some cases leading to a net power coproduct sold back to the grid. All financial, capital/operating cost, and design details utilized in the sugar model TEA for the pertinent operations in this modeled process are consistent with a previously published work.^25^

### Life Cycle Assessment

Life cycle assessment (LCA) on greenhouse gas (GHG) emissions and fossil energy consumption (FEC) was performed to evaluate and compare the GHG emissions and FEC associated with the sugar production TEA models described above at different disc refining operation conditions. LCA enables research to implement changes to modify and improve technologies, resulting in a more environmentally friendly product or process than otherwise might be produced. The scope of the LCA study focused on cradle-to-gate life cycle GHG emissions in kilograms of carbon dioxide equivalent (CO_2_e) using a 100 year GHG emission factor and FEC in MJ. The GHG and FEC factors were obtained from the 2021 version of Greenhouse gases, Regulated Emissions, and Energy use in Transportation (GREET) model^27^ and complemented with the Ecoinvent database^28^ for emission factors that were unavailable in GREET.

The corresponding reference flows and life-cycle inventories are summarized in Table 2, which are based on material and energy inputs and outputs to and from the biorefinery estimated from the Aspen-simulated conversion processes. The material and energy flows in the conversion step capture the impacts of input raw materials and outputs, such as emissions, wastes, and coproducts, as predicted by the process model. The coproducts (excess electricity in some cases) are treated as avoided products using the product displacement method.^29^ The processes did not require natural gas supplementation in the boiler, and the boiler flue gas CO_2_ emission in its entirety is biogenic CO_2_ (originated from biomass), which was excluded in the GHG accounting according to the IPCC methodology.^30^

**Table 2:** Life Cycle Inventory for the Sugar Production Models at Different Disc Refining Conditions with the Case Number Corresponding to the Experiments Shown in Table 1

case	1	2	3	4	5	6	7
products	production rate (kg/h)						
sugar production rate	38213	34323	30465	37983	38026	33981	31639
glucan/xylan conversion (%)^a^	84/79	77/68	69/58	85/77	85/77	78/63	80/45
carbon conversion (%)^b^	43	39	35	43	43	39	36
coproducts export electricity (kW)						1166	3356
resource consumption	flow rate (kg/h)						
biomass feedstock (20% moisture)	104167	104167	104167	104167	104167	104167	104167
sulfuric acid, 93%	8879	8879	8879	8879	8879	8879	8879
caustic (as pure)	6667	6667	6667	6667	6667	6667	6667
ammonia	604	604	603	604	604	603	603
flocculant	416.20	489.16	561.66	420.43	419.57	495.35	538.60
glucose	1986	1986	1986	1986	1986	1986	1986
corn steep liquor	135	135	135	135	135	135	135
corn oil	10.97	10.97	10.97	10.97	10.97	10.97	10.97
host nutrients	55.31	55.31	55.31	55.31	55.31	55.31	55.31
sulfur dioxide	13.48	13.48	13.48	13.48	13.48	13.48	13.48
diammonium phosphate	0.00	0.00	0.00	0.00	0.00	0.00	0.00
boiler chemicals	0.28	0.28	0.28	0.28	0.28	0.28	0.28
FGD lime	119.84	121.66	123.50	119.95	119.93	121.81	122.91
cooling tower chemicals	2.25	2.38	2.51	2.26	2.26	2.39	2.47
makeup water	232833	243235	255031	233120	233117	244134	250689
grid electricity (kW)	6257	2864	2880	1068	14031		
waste streams	flow rate (kg/h)						
disposal of brine	20914	20927	20946	20915	20915	20928	20940
disposal of ash	4418	4436	4455	4419	4419	4438	4449
air emissions	flow rate (kg/h)						
H_2_O	96817	105790	114735	97336	97229	106552	111873
N_2_	375185	391471	407675	376150	375957	392909	402748
CO_2_ (biogenic)	79916	85521	91082	80247	80184	86015	89391
O_2_	52560	53422	54292	52612	52599	53499	54028
NO_2_	66.95	72.15	77.31	67.26	67.20	72.61	75.74
SO_2_	7.21	7.32	7.43	7.21	7.21	7.33	7.39
CO	66.95	72.15	77.31	67.26	67.20	72.61	75.74
CH_4_	1.74	1.74	1.75	1.74	1.74	1.75	1.75

a Arabinan-to-arabinose conversion through EH held constant at 51% (arabinan conversion data were not collected here, but were fixed consistent with a previous work).

b Carbon conversion from biomass feedstock to the final sugar product.

## Computational Methods

### High-Fidelity Computational Geometric Domain

**Figure 2:**
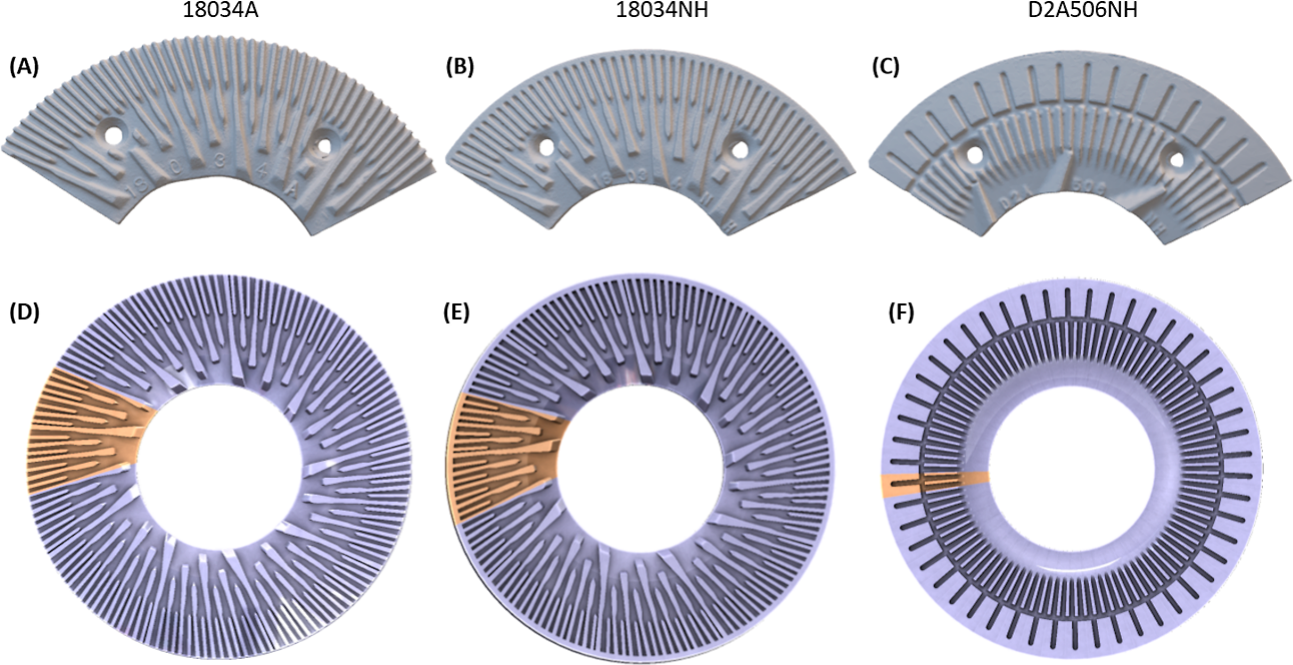
Refiner plates (18034A, D2A506NH, and 18034NH) used in experiments (A–C) and the corresponding computational geometry (D–F), respectively. The yellow-colored slice of the refiner plates in (D–F) is the smallest repeating unit and will be used as the computational domain in CFD simulations. Note the closed rim of plate 18034NH compared with 18034A.

Accurately defining the simulation geometric domain is another crucial aspect in the development of a high-fidelity model. In this work, a 3D laser scanner (NextEngine, model 2020i, Santa Monica, CA, USA) was used to prepare a blueprint model of various refiner plates. Based on the 3D scanned model of the plate, axisymmetric computational domains were developed, based on the 12 in. disc refiner described in the disc refining section above, using SolidWorks software to reduce computational complexity. Only one section of the whole disc refiner plate is used in the simulation by utilizing periodic boundary conditions without sacrificing computational fidelity, rendered with yellow color in Figure 2. More detailed information in preparing the model for simulation with extensive experimental validation of this approach has been reported in our previous work.^19^

### Governing Equations and Boundary Conditions

The mass and momentum conservation equations of the biomass slurry are solved in a moving reference frame after Reynolds averaging. These two equations are eqs 6 and 7, respectively.^31^ In this study, the moving reference frame is the coordinate system defined relative to the rotor (the moving part of a disc refiner). Alao, the stationary reference frame is defined relative to the refiner body. Relative velocity (**U**_**r**_) is the velocity of biomass slurry computed based on the moving reference frame. Absolute velocity (**U**) is computed based on the stationary reference frame. At any location in the computational domain, the velocity in the moving frame relative to the stationary frame (**V**_r_) is computed from the translational velocity (**U**_**t**_) and the angular velocity (**Ω**), as shown in eq 8. The shear–stress transport *k*–ω model^32^ was used to compute the Reynolds stress tensor (**R̅**) in eq 7. The viscous stress tensor (**τ̅**) in momentum conservation eq 7 is computed using eq 9. The viscosity (μ) in eq 7 is computed according to the HB model we parametrized in rheology measurements, eqs 4 and 5, based on the local shear rate, which is computed as the magnitude of the velocity gradient. Net refining energy consumption can be directly computed from the simulation predicted torque on the rotor. The torque is calculated based on the forces exerted on the rotor wall: the normal pressure force (**F**_p_) and the tangential viscous force (**F**_v_) shown in eqs 10 and 11, respectively. Then, with the measured idle energy consumption, total energy consumption and total specific energy consumption can be calculated from simulation results based on eqs 2 and 3, respectively.
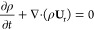
6
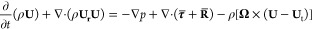
7
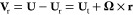
8
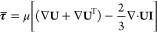
9
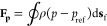
10
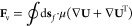
11

The outlet boundary is where the slurry exits the computational domain. At a certain location of the outlet boundary, the mixed boundary condition is used. If the flow is out of the domain, then a zero-gradient boundary condition is applied. For locations with the flow into the domain, a velocity computed based on the flux in the patch-normal direction is applied. Based on experiment parameters, a constant volumetric flow rate boundary condition at 3 L/min is used for the inlet boundary condition. No-slip boundary conditions are applied to the rotor and stator walls. A cyclic arbitrary mesh interface was used to couple those two periodic boundaries, which describes the rotational axisymmetric characteristics of the computational domain. The model was implemented using OpenFOAM. Simulations were performed using 64 cores on NREL’s Eagle high-performance computing system according to conditions listed in Table 1.

## Results and Discussion

### Effect of the Plate Gap on the Energy Consumption and Sugar Yield

**Figure 3:**
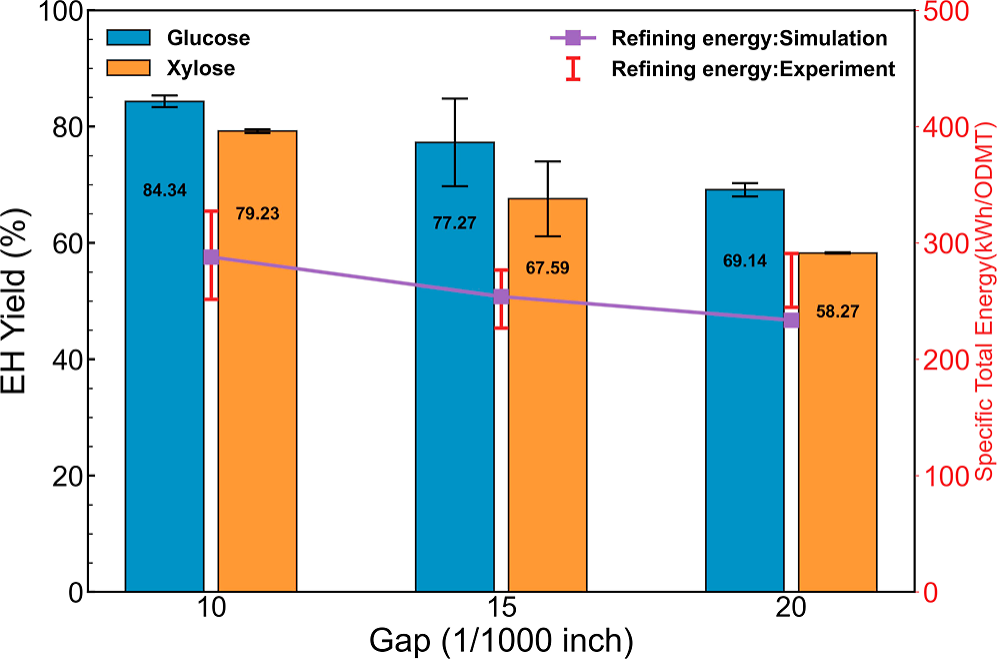
Total specific energy consumption and EH sugar yields of biomass slurry disc refined with plate 18034A at 900 rpm and different plate gaps.

A plate gap plays a critical role in the biomass disc refining process.^19,20^ We refined the deacetylated corn stover slurry using plate 18034A at 900 rpm with plate gaps of 10/1000, 15/1000, and 20/1000 in. The yield of sugars from EH of these disc refined corn stover cases is shown as a bar chart in Figure 3. Error bars on the bar chart plot represent the low and high range values of duplicated EH experiments. Experimentally measured and simulation predicted total specific energy consumptions at these refining conditions are shown as red error bars and the symbol-line plot in Figure 3. The red error bars represent the standard deviation of measured energy consumptions for the disc refining process over 5 min with a data collection speed of one sample per second. Glucose yield decreased from 84 to 69%, and xylose yield decreased from 79 to 58% when the plate gap was increased from 10/1000 to 20/1000 in. Experimentally measured specific total energy consumption shows a decreasing trend when the plate gap is increased. The simulation predicted specific total energy consumption agrees with experimental measurements decreasing from 288 kW h/ODMT to 234 kW h/ODMT at a plate gap of 0.01 and 0.2 in., respectively. Similar decreasing trends have been reported from the literature studying the disc refining of wood chips and pulping as well as our prior work studying the corn stover disc refining process using plate D2A506NH.^19^ We did observe that the sugar yields decrease proportionally to the specific total energy consumption, as this has been reported from prior works for woody biomass.^20,21^ As our prior work noted, as the plate gap is increased, the overall shear rate between refiner plates decreases, weakening the interaction between the plate and biomass slurry. This leads to less significant fiber disruption and thus lower sugar yield.

### Effect of Rotation Speed on Energy Consumption and Sugar Yield

**Figure 4:**
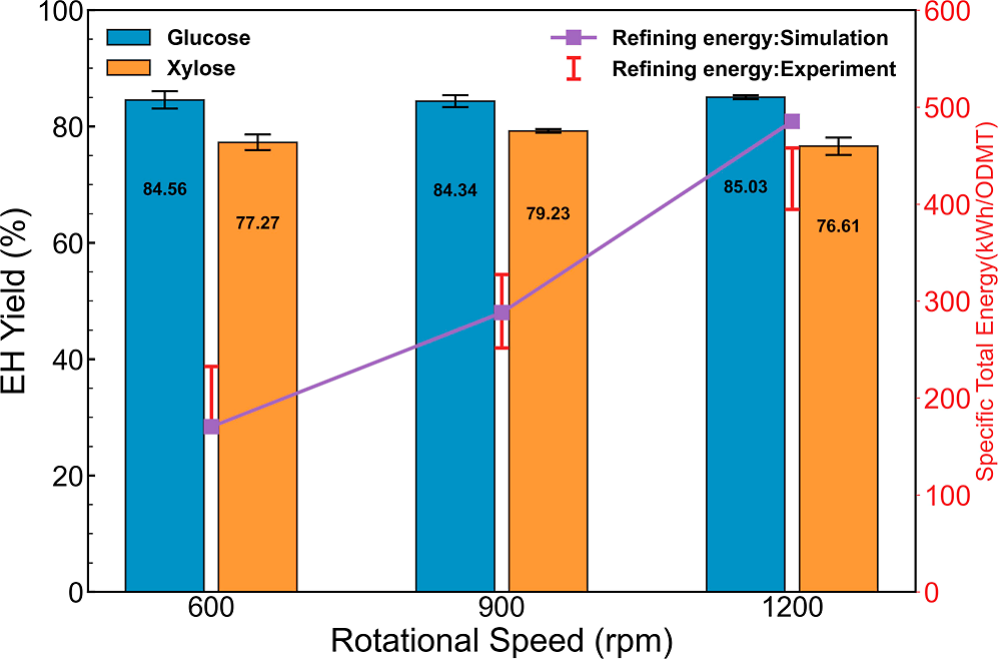
Total specific energy consumption and EH sugar yields of biomass slurry disc refined with the plate 18034A at 10/1000 in. gap and different rotation speeds.

It is known that the rotational speed directly impacts the refining intensity in the pulping process. However, most previous studies focus on studying the impact of plate gap, refining temperature, and refining solids consistency on the enzymatic sugar yields after disc refining.^20,21^ Rotation speed is generally overlooked and rarely reported as a point of interest in studying the disc refining process for biofuel applications. In this work, we performed disc refining of deacetylated corn stover slurry using plate 18034A with 10/1000 in. gap at different rotation speeds of 600, 900, and 1200 rpm. The effect of changing rotation speed on specific total energy consumption and enzymatic sugar yields is shown in Figure 4. As expected, increasing the rotation speed drastically increased the specific total energy consumption. From 600 to 1200 rpm, the specific total energy consumption increased by more than twice (201 to 426 kW h/ODMT). Surprisingly, we did not observe changes in the enzymatic sugar yields. Our previous modeling work showed that increasing rotational speed would increase the shear rate of the biomass slurry flowing between refiner plates. We believed that a higher rotational speed would create a stronger biomass–plate interaction. Also, the higher circulation flow velocity will also assist the formation of flocs and the subsequent disruption of lignocellulosic fibers. However, in this study, we did not observe any benefit from increasing the rotation speed. This is likely because even at 600 rpm and a 0.01 in plate gap, good enzymatic sugar yields were achieved (glucose yield of 85% and xylose yield of 77%), leaving little room for improvement at higher specific energy inputs. It is worth noting that this observation may be an isolated event and only applies to the conditions used in this work, which is a low consistency deacetylated corn stover slurry. Additionally, larger plate gaps may lead to different results. Without further experimental confirmation, conclusions from this set of experiments cannot be directly applied to other experimental conditions.

### Effect of Plate Geometry on Energy Consumption and Sugar Yield

**Figure 5:**
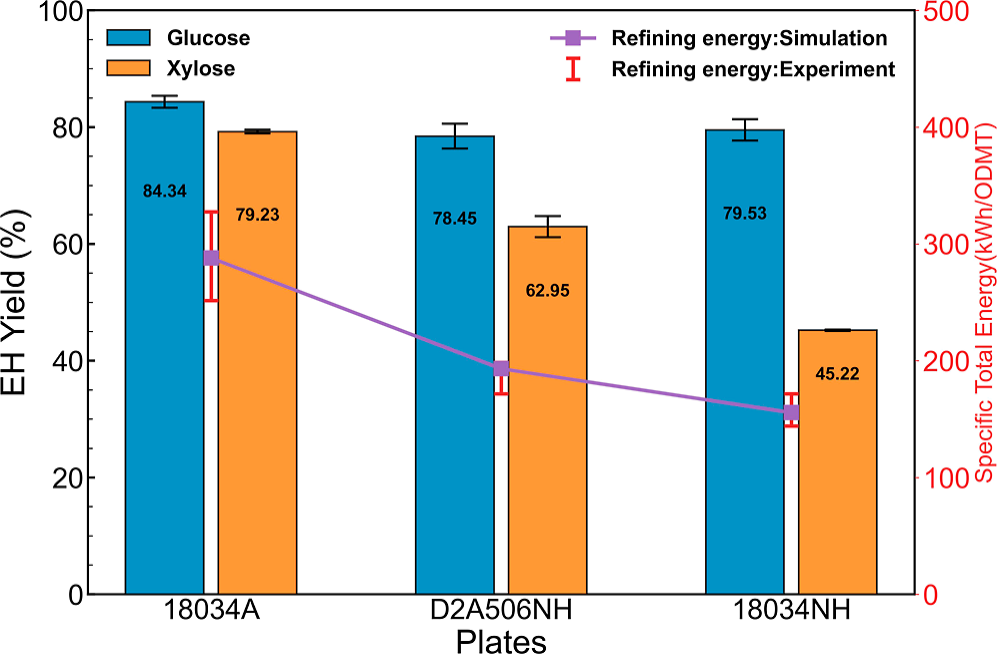
Total specific energy consumption and EH sugar yields of biomass slurry disc refined at 900 rpm and 10/1000 in. gap with different plates.

There are ongoing efforts to understand how plate geometric design can improve pulp quality and reduce energy consumption in the pulping research area. Research on how plate design can affect disc refining in lignocellulosic biofuel production processes is rare. For the lignocellulosic biofuel to be successful (pathways with mechanical refining step(s), e.g., the DMR pretreatment process), understanding the effect of the refiner plate geometry on sugar yields is critical for process optimization. We performed disc refining of deacetylated corn stover using three different refiner plates while keeping other operating conditions constant at a rotation speed of 900 rpm with 10/1000 in. gap. These three plates are shown in Figure 2 and labeled 18034A, D2A506NH, and 18034NH. Plates 18034A and 18034NH are the same for the most part, except that plate 18034NH has an island structure at the outer rim of the plate, while plate 18034A does not. Plate D2A506NH is a completely different design compared to 18034A and 18034NH. Plate D2A506NH has a much wider dam (or can be referred to as an island) with an increasing intergroove distance from the center to the edge. The effects of refiner plate design on specific total energy consumption and enzymatic sugar yields are shown in Figure 5.

We observed that at the same refining operating conditions, refiner plates D2A506NH and 18034NH had significantly lower specific total refining energy consumption (from 290 to 185 and 158 kWh/ODMT) than 18034A. Our model was able to accurately predict the energy consumption for different plates, proving that the CFD base model is able to predict specific energy consumption for different plate designs. This is an advance over the existing empirical and traditional disc refiner models because of its wide applicability to different plate designs and the possibility of being used to optimize these designs.

We normally expect the enzymatic sugar yields to be positively correlated to specific total energy consumptions of the disc refining process. Also, this conclusion has been reported in many reports studying the EH of disc refined lignocellulosic biomass,^9,20^ where specific total energy consumption is used as a key parameter to represent refining intensity. However, our results show interesting trends that warrant a deeper look at the disc refining process. We observed that glucose yield only slightly decreased (from 84 to 78 and 80%) when changing from plate 18034A to plate D2A506NH and plate 18034NH while observing an almost proportional decrease in xylose yield. The impact of changing plates on enzymatic sugar yields is significantly different for glucose and xylose. Glucose yield decreased by 7 and 6%, while xylose yield decreased by 21 and 44%, and specific total energy consumption decreased by 36 and 45% by changing plates from 18034A to D2A506NH and 18034NH, respectively. The result clearly indicates that by changing the plate design, the electricity consumption can be decreased while maintaining relatively high glucose yields.

### Biomass Slurry Flow Behavior Affected by Plates

**Figure 6:**
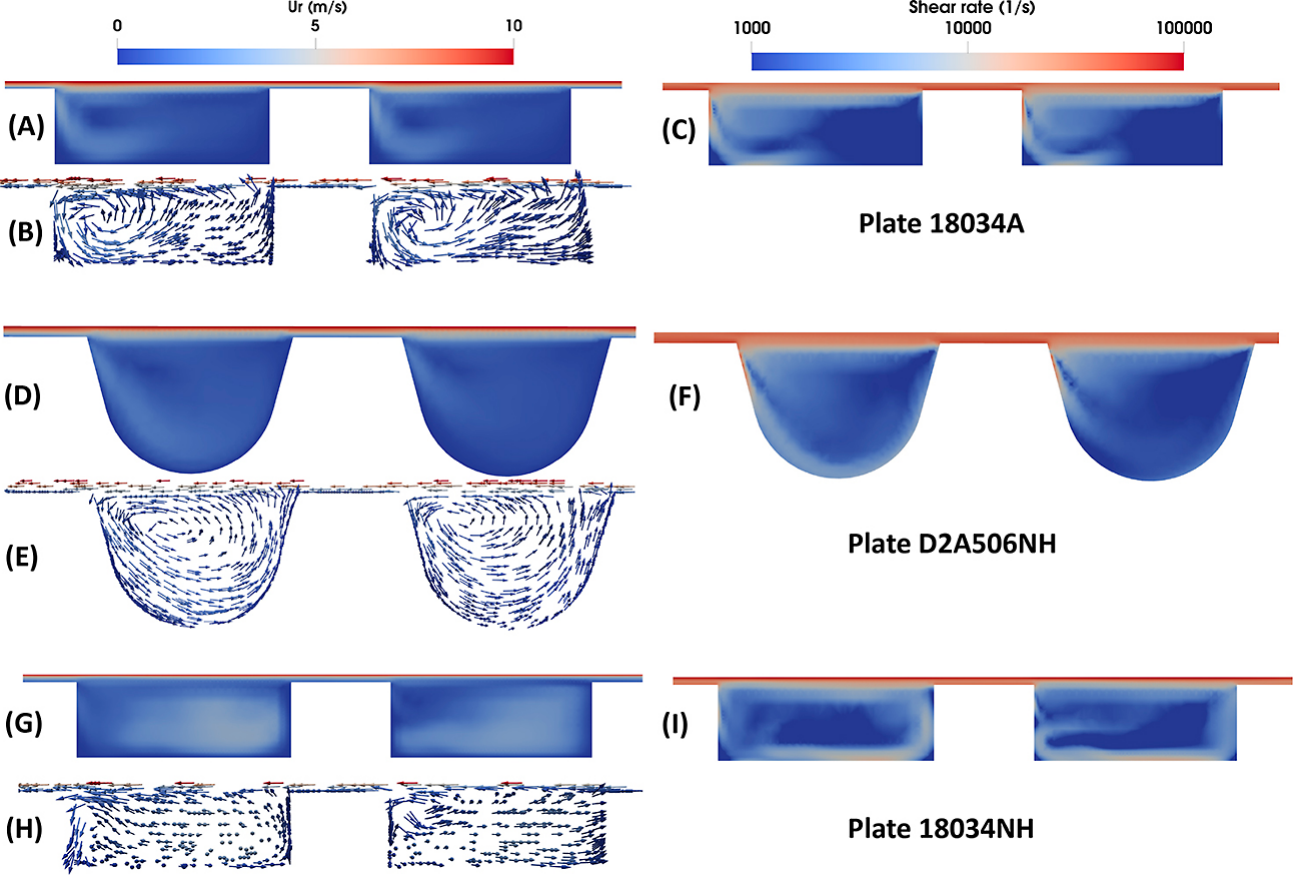
Comparison of the slurry flow and shear rate at radial location of 4.5 in. inside disc refiners using three different plates: (A–C): plate 18034A; (D–F): plate D2A506NH; and (G–I): plate 18034NH. (A,D,G) Velocity magnitude distributions; (B,E,H) slurry flow vector maps using constant arrow length colored by velocity magnitude; and (C,F,I) shear rate distributions.

To further understand the effect of plate geometry on the disc refining process, we examined the flow velocity and shear rate distribution at *R* = 4.5 in. with flow direction facing outside of the paper, as shown in Figure 6. Comparing Figure 6A,G, we see that with the addition of a dam or a closed barrier at the outer rim of the refiner plate (18034NH), the fluid flow inside the grooves is relatively more uniform before reaching the obstacle than the very similar plate but without an outer rim barrier structure (18034A). For plate D2A506NH, though it has a completely different design, the flow inside the grooves also shows better uniformity than plate 18034A. All three plates show that there is a circulation flow pattern formed close to the groove side wall, the one which is pushing the slurry, as seen in Figure 6B,E,H. Coupled with the results shown in Figure 6A,D,G, the circulation intensity generated by plate 18034A is the strongest followed by plate D2A506NH and then plate 18034NH. If we look at the shear rate distributions in Figure 6C,F,I, higher circulation intensity generates a higher shear rate around the slurry shearing edge of groove. Based on these simulation results and the enzymatic sugar yields of the biomass slurry refined using these three plates, we observed a positive correlation between circulation flow intensity and shear rate with the enzymatic sugar yields. This may be attributed to the fact that the strong circulation flow inside the grooves aids the formation of biomass fiber entanglement and the disruption of biomass fibers.

### Effect of Disc Refining on Minimum Sugar Selling Price

**Figure 7:**
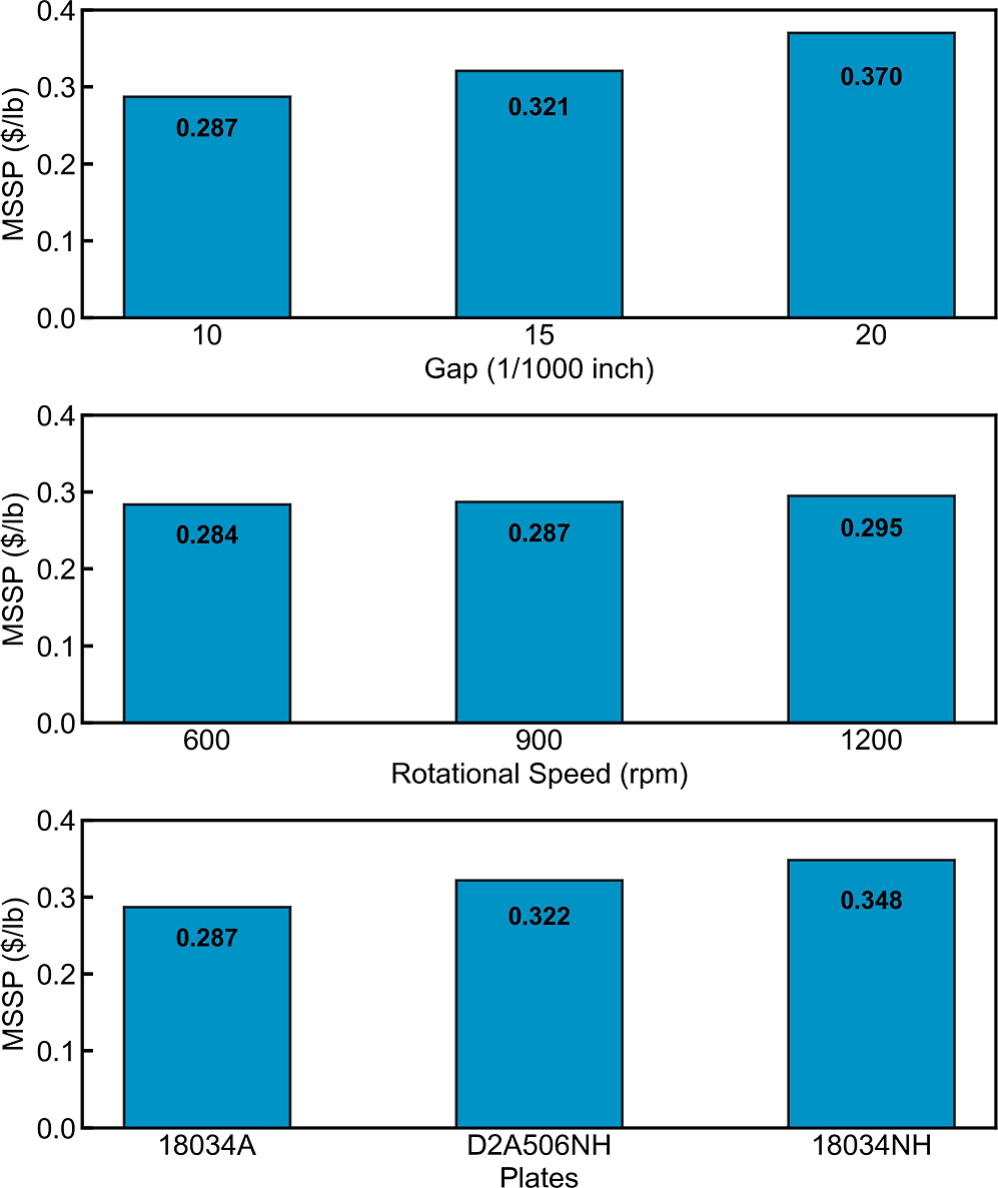
Impact of different disc refining conditions on the minimum sugar selling price: plate gap (top) operated at 900 rpm with plate 18034A, rotational speed (middle) operated using plate 18034A with 10/1000 in. plate gap, and plate geometric pattern (bottom) operated at 900 rpm with 10/1000 in. plate gap.

Upon evaluation of these cases through TEA modeling, it was found that increasing the plate gap results in a higher minimum sugar selling price (MSSP) despite the lower power consumption at higher plate gaps, as shown on the top graph of Figure 7. Though the power consumption decrease is correlated to the decrease in sugar yields, the cost reduction from lowering power consumption by increasing the plate gap is not able to offset the loss in revenue from the reduced sugar yield. This indicates that when optimizing the plate gap, sugar yields have a higher impact on MSSP than power consumption. Reducing rotational speed results in lower MSSP, shown in the middle graph of Figure 7, as the sugar yields remained almost unchanged, while power consumption is significantly decreased at lower rotational speeds. Comparing different plate geometries, using plate 18034A design results in the lowest MSSP among those three plate patterns, as shown in the bottom graph in Figure 7. With glucose yields kept almost the same, the proportionate decrease in xylose yield with respect to refining energy consumption dominates the impact on MSSP when comparing different plate patterns (assuming that the disc refiner capital cost is unchanged for different plate designs). These results emphasize that sugar yields remain the most impactful driver in lowering the minimum sugar selling price for the DMR process. The optimization of the disc refining process should maintain high sugar yields while decreasing its energy consumption to achieve lower MSSP.

It is recognized that not all hydrolysate sugars are equally fermentable depending on the downstream organism/process. The sugar yield and cost metrics calculated in the NREL TEA sugar model include the three primary hydrolysate sugar monomers—glucose, xylose, and arabinose—although minimal amounts of galactose and mannose are also usually present in typical hydrolysates at low concentrations that are not often tracked through fermentation. Glucose is produced at a roughly 2:1 ratio versus xylose and together constitute roughly 98% of the total sugar yield calculated in the TEA models (with arabinose present in the models on the order of 2%). While recognizing that these three sugars are not necessarily equally valuable to a fermentation biorefinery processor as glucose is often more readily convertible than xylose and in turn arabinose (and accordingly the relative *value* of these sugars would follow a similar order), for TEA purposes, it would only make sense to differentiate the production *cost* of individual sugars if they were being split into separate downstream processing trains, which is not often pursued as it incurs significant additional costs and biorefinery complexity. Instead, the fermentation processor must more optimally tailor fermentation conditions or strain engineering toward effectively utilizing all of the major hydrolysate sugars, particularly glucose and xylose, to maximize downstream yields and minimize costs.

Table 3 provides further details on key TEA model outputs, including MSSP, sugar yields, and power demands for the disc refiner and overall facility (prior to accounting for offsets from power generation). As can be seen in the table, case 4 (plate 18034A; 10/1000 in. gap; 600 rpm) gives the best performance in terms of the minimum sugar selling price, with cases 1 and 5 (900 and 1200 rpm) trailing slightly behind. Case 4 also had good performance in terms of minimizing the disc refiner and overall facility power usage, which were considerably worse for cases 1 (900 rpm) and 5 (1200 rpm), with the latter representing a substantially higher power demand than all other cases evaluated.

**Table 3:** Minimum Sugar Selling Prices for Each Case along with Sugar Yield, Power Demands (Prior to Accounting for Power Generation from Lignin Combustion), and the Fraction of Total Facility Power Demand Attributed to Mechanical Refining

case	MSSP ($/lb)	sugar yield (lb/ton)	biorefinery electricity use (kW)		
			whole plant	mechanical refining	percentage (%)
1	0.287	917.1	48878	16691	34
2	0.321	823.8	48141	14521	30
3	0.370	731.2	50966	15440	30
4	0.284	911.6	43856	11609	26
5	0.295	912.6	56803	24567	43
6	0.322	815.5	44378	10675	24
7	0.348	759.3	43869	9109	21

### Effect of Disc Refining on Carbon Emissions and Fossil Energy Consumption

**Figure 8:**
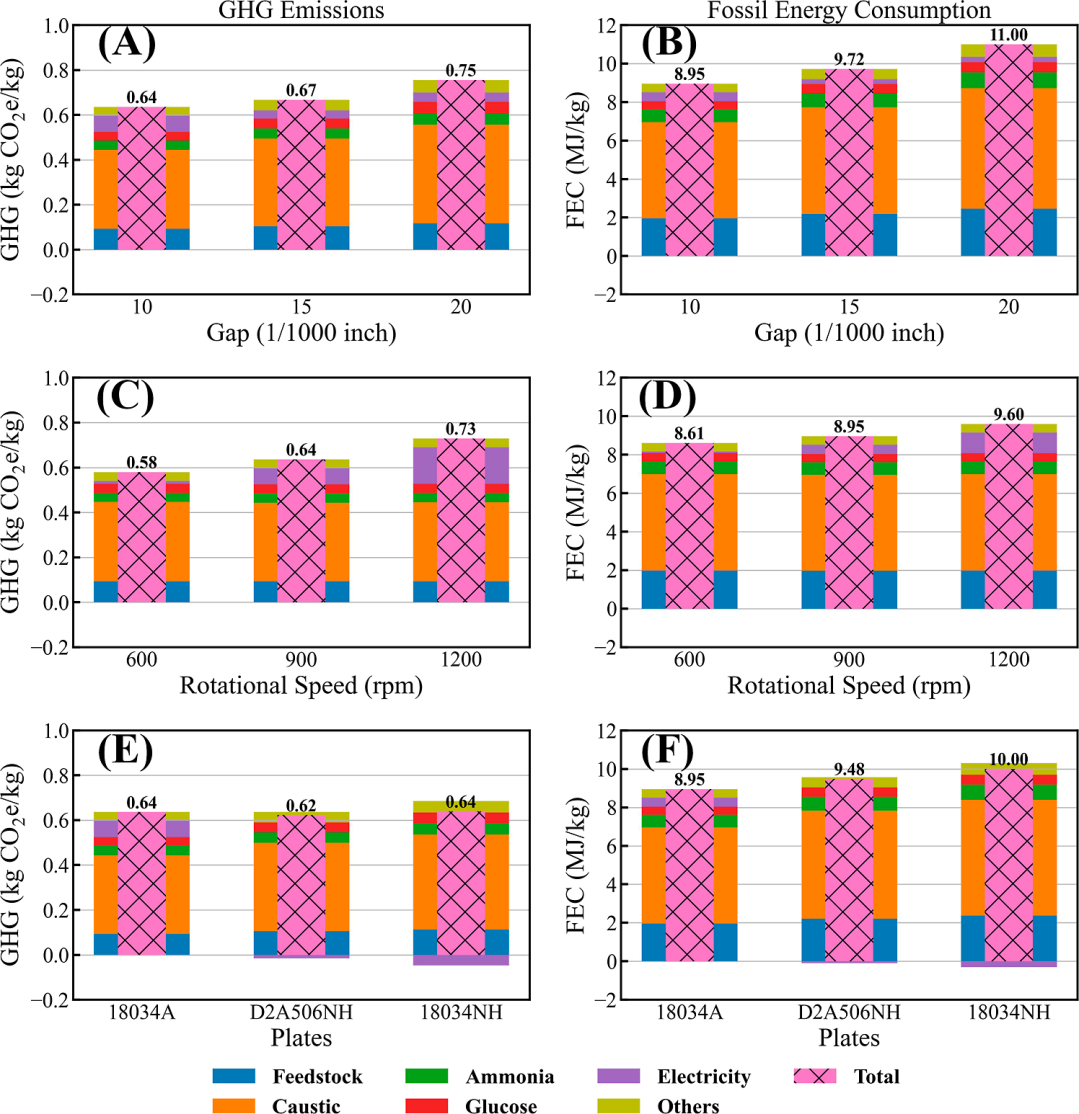
Impact of different disc refining operation conditions on the GHG emissions (A,C,E) and fossil energy consumption (B,D,F) as a function of plate gap (A,B) operated at 900 rpm with plate 18034A, rotational speed (C,D) operated using plate 18034A with 10/1000 in. plate gap, and plate geometric pattern (E,F) operated at 900 rpm with 10/1000 in. plate gap.

Figure 8 shows that increasing the plate gap from 10/1000 to 20/1000 in. results in higher GHG emissions, from 0.64 to 0.75 kg of CO_2_e/kg of sugar, respectively. The GHG emission profiles correlate well with those of the fossil energy consumption (FEC). Caustic (sodium hydroxide) and biomass feedstock inputs are the two largest contributors to GHGs and FEC. Additionally, increasing the plate gap led to lower sugar yields and corresponding decreasing power demands (Table 2, Cases 1–3), resulting in higher specific power consumption per sugar production output (and higher GHGs and FEC). Similar to the TEA, when optimizing the plate gap, sugar yields exhibit a higher impact on GHGs and FEC than power consumption alone, although power consumption is a stronger driver on LCA than on TEA.

On the other hand, reducing rotational speed results in lower GHGs and FEC, shown in the middle graph of Figure 8, as the sugar yields were similar, but power consumption significantly decreased as discussed previously (rotational speed/power consumption): 600 rpm/1068 kW < 900 rpm/6257 kW < 1200 rpm/14,031 kW (Table 2, Cases 4, 1, and 5, respectively).

Comparing different plate geometries, using plate 18034A design results in the lowest MSSP and FEC, but the D2A506NH design exhibits the lowest GHGs, as shown in the bottom graph in Figure 8. Both D2A506NH and 18034NH designs consume significantly less power than the 18034A design (Table 2, Cases 6, 7, and 1, respectively), leading to a net power coproduct export to the grid from the overall facility for those two cases. However, the excess electricity coproduct credits for both the D2A506NH and 18034NH designs could not compensate for their relatively lower sugar yields in the cost results presented above.

The LCA results indicate that the plate design and operating conditions have a direct impact on the process power consumption and sugar yields. It is sugar yields that predominantly dictate the life cycle GHG emissions and FEC, though power consumption also exhibits stronger drivers for LCA than TEA. Among all the scenarios, Case 3 (20/1000-in. plate gap) demonstrates the highest GHGs (0.75 kg CO_2_e/kg) and FEC (11.0 MJ/kg), and Case 4 (600 rpm) shows the lowest GHGs (0.58 kg CO_2_e/kg) and FEC (8.61 MJ/kg). Thus, Case 4 (10/1000 in. plate gap, 600 rpm rotational speed, and 18034A plate design) represents the most favorable disc refining process, maintaining high sugar yields while decreasing energy consumption to achieve favorable GHGs and FEC, as well as MSSP. While a lower rotational speed could likely further improve the GHG results for the D2A506NH design (i.e., 600 rpm, not evaluated in this experimental set), the GHG savings between the 18034A and D2A506NH design (roughly 3% GHG reduction) are not enough to justify the disproportionately higher sugar cost (roughly 12% higher MSSP); thus, the combination of parameters in Case 4, plate gap of 10/1000 in. with 600 rpm rotational speed, for the 18034A design lead to the overall optimal configuration considering both TEA and LCA trade-offs together.

## Conclusions

Reducing enzymatic sugar costs and the subsequent biofuel price, as well as corresponding GHG intensity, is a key challenge for mechanical refining-based pretreatment technologies. The positive correlation between energy consumption and enzymatic sugar yields for any disc refining condition may initially appear straightforward. In this study, we found that for low consistency disc refining of mild alkaline pretreated corn stover, the enzymatic sugar yields exhibit a positive correlation with specific energy consumption at different refiner gaps, which agrees with conclusions reported in the literature. However, we observed that changing the rotational speed proportionally changed the energy consumption without impacting the enzymatic sugar yields under experimental conditions in this study. This indicates that, at current conditions, over-refining biomass does not benefit the EH process. We also demonstrated that the refiner plate geometry has a significant impact on the relationship between enzymatic sugar yields and specific energy consumption. We showed that a 45% reduction in specific energy consumption can be achieved without affecting glucose yield by using different refiner plates though still impacting xylose yield. Further study is needed to elucidate how changing plate geometry can have different effects on glucose and xylose yield.

Our computational model demonstrated an accurate prediction of specific energy consumption from the disc refining process simulations compared to experimental measurements. The simulation results revealed that refiner plates with a dam structure at the outer rim, which have low energy consumption, generate less severe circulation flow inside the grooves, and lower the shear rate around the slurry shearing edge. Our model showed potential in helping to optimize the refining parameters and refiner plate geometry to achieve the desired flow patterns and shear in the disc refining process. TEA and LCA modeling showed that sugar yields remain the strongest driver in lowering the minimum sugar selling price, GHG emissions, and fossil energy consumption for the DMR process, which should apply to similar pretreatment technologies based on mechanical refining; however, power demands also exhibit an outsized influence on LCA performance more so than TEA performance. The optimization of disc refining conditions for lower MSSP and more favorable environmental sustainability metrics should maintain high sugar yields while decreasing the energy consumption.

## Abbreviations

CFD: computational fluid dynamics
DMR: deacetylation and mechanical refining
GHG: greenhouse gas
FEC: fossil energy consumption
HB: Herschel–Bulkley
INL: Idaho National Laboratory
NREL: National Renewable Energy Laboratory
RPM: revolutions per minute
MSSP: minimum sugar selling price
TEA: techno-economic analysis
LCA: life cycle assessment

## Latin Symbols

*k*: constant in Herschel–Bulkley model
*n*: constant in Herschel–Bulkley model
*P*_idle_: idle energy consumption (kW)
*P*_net_: net energy consumption (kW)
*P*_total_: total energy consumption (kW)
*e*_*i*_: *i* = *net* or *shear*; specific energy (kJ/kg)
*ṁ*_b_: biomass feed rate (kg/s)
*t*: time (s)
**U**_r_: relative velocity vector
**U**: absolute velocity vector
**U**^T^: transpose of absolute velocity vector
**U**_t_: translational velocity vector
**V**_r_: velocity of the moving frame vector
*R̅*: Reynolds stress tensor
**r**: distance vector
**I**: identity matrix
*p*: pressure (Pa)
*p*_ref_: reference pressure (Pa)
sf: face area vector
**F**_p_: normal pressure force vector
**F**_v_: tangential viscous force vector

## Greek Symbols

α: constant in idle power model
β: constant in idle power model
τ: shear stress (Pa)
τ_y_: yield stress (Pa)
γ*˙*: shear rate (1/s)
μ: viscosity (Pa·s)
ρ: density (kg/m^3^)
Τ*®*: viscous stress tensor
Ω_R_: rotation speed of rotor (RPM)
**Ω**: angular velocity vector

## References

[ref1] Wyman C. E.; Dale B. E.; Elander R. T.; Holtzapple M.; Ladisch M. R.; Lee Y. Y. Coordinated Development of Leading Biomass Pretreatment Technologies. Bioresour. Technol. 2005, 96 (18), 1959–1966. 10.1016/j.biortech.2005.01.010. 16112483

[ref2] Calcio Gaudino E.; Cravotto G.; Manzoli M.; Tabasso S. From Waste Biomass to Chemicals and Energy via Microwave-Assisted Processes. Green Chem. 2019, 21 (6), 1202–1235. 10.1039/C8GC03908A.

[ref3] Rajak R. C.; Saha P.; Singhvi M.; Kwak D.; Kim D.; Lee H.; Deshmukh A. R.; Bu Y.; Kim B. S. An Eco-Friendly Biomass Pretreatment Strategy Utilizing Reusable Enzyme Mimicking Nanoparticles for Lignin Depolymerization and Biofuel Production. Green Chem. 2021, 23 (15), 5584–5599. 10.1039/D1GC01456K.

[ref4] Chen X.; Kuhn E.; Jennings E. W.; Nelson R.; Tao L.; Zhang M.; Tucker M. P. DMR (Deacetylation and Mechanical Refining) Processing of Corn Stover Achieves High Monomeric Sugar Concentrations (230 g L-1) during Enzymatic Hydrolysis and High Ethanol Concentrations (>10% v/v) during Fermentation without Hydrolysate Purification or Concentration. Energy Environ. Sci. 2016, 9 (4), 1237–1245. 10.1039/C5EE03718B.

[ref5] Li Y.; Chen X.; Sievers D. A. Modelling a Compressible Packed Bed Flow-through Washing and Deacetylation Reactor for Corn Stover Pretreatment. Chem. Eng. J. 2021, 415, 128918 10.1016/j.cej.2021.128918.

[ref6] Kuhn E. M.; Chen X.; Tucker M. P. Deacetylation and Mechanical Refining (DMR) and Deacetylation and Dilute Acid (DDA) Pretreatment of Corn Stover, Switchgrass, and a 50:50 Corn Stover/Switchgrass Blend. ACS Sustain. Chem. Eng. 2020, 8 (17), 6734–6743. 10.1021/acssuschemeng.0c00894.

[ref7] Chen X.; Shekiro J.; Franden M. A.; Wang W.; Zhang M.; Kuhn E.; Johnson D. K.; Tucker M. P. The Impacts of Deacetylation Prior to Dilute Acid Pretreatment on the Bioethanol Process. Biotechnol. Biofuels 2012, 5, 8 10.1186/1754-6834-5-8. 22369467 PMC3309953

[ref8] Chen X.; Wang W.; Ciesielski P.; Trass O.; Park S.; Tao L.; Tucker M. P. Improving Sugar Yields and Reducing Enzyme Loadings in the Deacetylation and Mechanical Refining (DMR) Process through Multistage Disk and Szego Refining and Corresponding Techno-Economic Analysis. ACS Sustain. Chem. Eng. 2016, 4 (1), 324–333. 10.1021/acssuschemeng.5b01242.

[ref9] Chen X.; Shekiro J.; Pschorn T.; Sabourin M.; Tao L.; Elander R.; Park S.; Jennings E.; Nelson R.; Trass O.; Flanegan K.; Wang W.; Himmel M. E.; Johnson D.; Tucker M. P. A Highly Efficient Dilute Alkali Deacetylation and Mechanical (Disc) Refining Process for the Conversion of Renewable Biomass to Lower Cost Sugars. Biotechnol. Biofuels 2014, 7 (1), 98 10.1186/1754-6834-7-98.

[ref10] Chen X.; Crawford N.; Wang W.; Kuhn E.; Sievers D.; Tao L.; Tucker M. Kinetics and Rheological Behavior of Higher Solid (Solids > 20%) Enzymatic Hydrolysis Reactions Using Dilute Acid Pretreated, Deacetylation and Disk Refined, and Deacetylation and Mechanical Refined (DMR) Corn Stover Slurries. ACS Sustain. Chem. Eng. 2019, 7 (1), 1633–1641. 10.1021/acssuschemeng.8b05391.

[ref11] Li B.; Li H.; Zha Q.; Bandekar R.; Alsaggaf A.; Ni Y. Review: Effects of wood quality and refining process on TMP pulp and paper quality. BioResources 2011, 6 (3), 3569–3584. 10.15376/biores.6.3.3569-3584.

[ref12] Rubiano Berna J. E.; Martinez M.; Olson J. Power-Gap Relationships in Low Consistency Refining. Nord. Pulp Pap. Res. J. 2019, 34 (1), 36–45. 10.1515/npprj-2018-0039. PMC10044004 37011240

[ref13] Kerekes R. J.; McDonald J. D. Bar Forces in Pulp Refiners. Nord. Pulp Pap. Res. J. 2021, 36 (4), 714–717. 10.1515/npprj-2021-0044.

[ref14] Elahimehr A.; Olson J. A.; Martinez D. M. Understanding LC Refining: The Effect of Plate Pattern and Refiner Operation. Nord. Pulp Pap. Res. J. 2013, 28 (3), 386–391. 10.3183/npprj-2013-28-03-p386-391.

[ref15] Elahimehr A.; Olson J. A.; Martinez D. M. Low Consistency Refining of Mechanical Pulp: How Plate Pattern and Refiner Operating Conditions Change the Final Properties of Pulp. Nord. Pulp Pap. Res. J. 2015, 30 (4), 609–616. 10.3183/npprj-2015-30-04-p609-616.

[ref16] Elahimehr A.; Olson J. A.; Martinez D. M.; Heymer J. MECHANICAL PULPING: Estimating the Area and Number of Bar Crossings between Refiner Plates. Nord. Pulp Pap. Res. J. 2012, 27 (5), 836–843. 10.3183/npprj-2012-27-05-p836-843.

[ref17] Wittberg L. P.; Björkman M.; Khokhar G.; Mohlin U.-B.; Dahlkild A. Flow Conditions in the Grooves of a Low-Consistency Refiner. Nord. Pulp Pap. Res. J. 2012, 27 (2), 173–183. 10.3183/npprj-2012-27-02-p173-183.

[ref18] Kondora G.; Asendrych D. Flow Modelling in a Low Consistency Disc Refiner. Nord. Pulp Pap. Res. J. 2013, 28 (1), 119–130. 10.3183/npprj-2013-28-01-p119-130.

[ref19] Li Y.; Sievers D. A.; Chen X. Modeling the Disc Refining of Lignocellulosic Biomass toward Reduced Biofuel Production Cost and Greenhouse Gas Emissions: Energy Consumption Prediction and Validation. ACS Sustain. Chem. Eng. 2021, 9 (29), 9717–9726. 10.1021/acssuschemeng.1c01773.

[ref20] Zhu W.; Zhu J. Y.; Gleisner R.; Pan X. J. On Energy Consumption for Size-Reduction and Yields from Subsequent Enzymatic Saccharification of Pretreated Lodgepole Pine. Bioresour. Technol. 2010, 101 (8), 2782–2792. 10.1016/j.biortech.2009.10.076. 20006490

[ref21] Jones B. W.; Venditti R.; Park S.; Jameel H. Optimization of Pilot Scale Mechanical Disk Refining for Improvements in Enzymatic Digestibility of Pretreated Hardwood Lignocellulosics. BioResources 2017, 12 (3), 4567–4593. 10.15376/biores.12.3.4567-4593.

[ref22] Sluiter A.; Hames B.; Ruiz R.; Scarlata C.; Sluiter J.; Templeton D.; Crocker D. Determination of Structural Carbohydrates and Lignin in Biomass. Technical Report NREL/TP-510–42618; National Renewable Energy Laboratory, 2008.

[ref23] McMillan J. D.; Jennings E. W.; Mohagheghi A.; Zuccarello M. Comparative Performance of Precommercial Cellulases Hydrolyzing Pretreated Corn Stover. Biotechnol. Biofuels 2011, 4 (1), 29 10.1186/1754-6834-4-29. 21899748 PMC3200994

[ref24] NREL 2017 Biochemical Sugar Model. https://www.nrel.gov/extranet/biorefinery/aspen-models/ (accessed Oct 2, 2022).

[ref25] Davis R. E.; Grundl N. J.; Tao L.; Biddy M. J.; Tan E. C.; Beckham G. T.; Humbird D.; Thompson D. N.; Roni M. S.. In Process Design and Economics for the Conversion of Lignocellulosic Biomass to Hydrocarbon Fuels and Coproducts: 2018 Biochemical Design Case Update; Biochemical Deconstruction and Conversion of Biomass to Fuels and Products via Integrated Biorefinery Pathways; National Renewable Energy Lab. (NREL): Golden, CO (United States), 2018.

[ref26] Chen X. A Transformational New Process Paradigm to Produce Low Cost Sugars from Biomass, 2014.

[ref27] Wang M. The Greenhouse Gases, Regulated Emissions, and Energy Use in Transportation (GREET) Model: Version 1.5; Center for Transportation Research Argonne National Laboratory, 2008.

[ref28] Wernet G.; Bauer C.; Steubing B.; Reinhard J.; Moreno-Ruiz E.; Weidema B. The Ecoinvent Database Version 3 (Part I): Overview and Methodology. Int. J. Life Cycle Assess. 2016, 21 (9), 1218–1230. 10.1007/s11367-016-1087-8.

[ref29] Tao L.; Tan E. C. D.; McCormick R.; Zhang M.; Aden A.; He X.; Zigler B. T. Techno-Economic Analysis and Life-Cycle Assessment of Cellulosic Isobutanol and Comparison with Cellulosic Ethanol and n-Butanol. Biofuels, Bioprod. Biorefin. 2014, 8 (1), 30–48. 10.1002/bbb.1431.

[ref30] IPCC. International Panel on Climate Change (IPCC) Fifth Assessment Report—Impacts, Adaptation and Vulnerability; Key Findings from the Intergovernmental Panel on Climate Change (IPCC) Fifth Assessment Report (AR5). Cambridge (UK); University of Cambridge, Institutional Investors Group on Climate Change and UNEP Finance Initiative, 2014.

[ref31] Ferziger J. H.; Perić M. Computational Methods for Fluid Dynamics; Springer, 2002; Vol. 3, pp 4–9.

[ref32] Menter F. R. Two-Equation Eddy-Viscosity Turbulence Models for Engineering Applications. AIAA J. 1994, 32 (8), 1598–1605. 10.2514/3.12149.

